# Reducing Metabolic Dysregulation in Obese Latina and/or Hispanic Breast Cancer Survivors Using Physical Activity (ROSA) Trial: A Study Protocol

**DOI:** 10.3389/fonc.2022.864844

**Published:** 2022-05-10

**Authors:** Paola Gonzalo-Encabo, Rebekah L. Wilson, Dong-Woo Kang, Mary K. Norris, Hajime Uno, Cami N. Christopher, Christina Chow, Nathalie Sami, Frank S. Fox, Jennifer A. Ligibel, Christina M. Dieli-Conwright

**Affiliations:** ^1^ Division of Population Sciences, Department of Medical Oncology, Dana-Farber Cancer Institute, Boston, MA, United States; ^2^ Department of Medicine, Harvard Medical School, Boston, MA, United States; ^3^ UCSF School of Dentistry, University of California, San Francisco, San Francisco, CA, United States; ^4^ Department of Internal Medicine, Los Angeles County-University of Southern California (LAC+USC) Medical Center, Keck School of Medicine, Los Angeles, CA, United States; ^5^ Gerson Lehrman Group, New York, NY, United States; ^6^ Division of Breast Oncology, Department of Medical Oncology, Dana-Farber Cancer Institute, Boston, MA, United States

**Keywords:** insulin resistance, adiposity, cancer minorities, aerobic exercise, resistance exercise

## Abstract

**Background:**

Latina and Hispanic breast cancer survivors (LHBCS) are at increased risk for long-term complications and poorer metabolic health, including metabolic dysregulation (MetD) before and following breast cancer diagnosis. MetD can increase risk of cancer recurrence, death, and comorbid conditions by increasing inflammation and cancer cell proliferation. While exercise improves physical fitness and metabolic outcomes in breast cancer survivors, there is a lack of studies including underrepresented and disadvantaged minority groups such as LHBCS.

**Methods:**

Our 12-month randomized (exercise or attention control) controlled trial (the ROSA trial) aims to utilize a progressive combined aerobic and resistance exercise program to improve MetD, insulin resistance, and visceral adiposity among obese LHBCS. We aim to recruit 160 women with Stage I-III breast cancer who are sedentary, centrally obese, and have completed treatment (e.g., surgery, radiation, chemotherapy) prior to enrollment. Participants randomized to the exercise group receive 16-weeks of virtually supervised aerobic and resistance training, followed by 16-weeks of unsupervised home-based aerobic and resistance exercise, and 16-weeks of follow-up. The attention control group receive a 12-month home-based stretching program. Primary and secondary outcomes are measured every 4-weeks during study visits.

**Discussion:**

The ROSA trial is the first exercise oncology trial targeting high-risk sedentary, obese LHBCS to improve MetD-related outcomes. Results of this trial will help illuminate how exercise impacts health-related outcomes, survivorship, and recurrence, and inform future exercise oncology guidelines to reduce health disparities among minority cancer survivors.

## 1 Introduction

In the United States (US), there are over 62 million people identifying as Latinx/a/o or Hispanic, comprising 18.7% of the nation’s total population in 2020 (1, 2). By 2060, this is expected to increase to 111.2 million, accounting for 28% of the total population of the US, making the Latinx/a/o and Hispanic population the largest and fastest-growing ethnic minority groups (1, 3, 4). Among Latina and Hispanic women in the US, breast cancer is the leading cancer diagnosed and the leading cause of cancer deaths (5). Latina and Hispanic women are at a higher risk of breast cancer mortality, advanced cancer stage at diagnosis, and poorer breast cancer prognosis when compared to non-Latina white women (6, 7). The terms Latinx/a/o and Hispanic are typically used to refer to persons with descent from Cuba, Mexico, Puerto Rico, South or Central America, or other Spanish culture or origins (1, 2). For the purpose of this manuscript and trial, we will use both Latina and Hispanic terms to refer to women with breast cancer with descent to previously mentioned cultures and origins.

One major health concern of particular interest among breast cancer survivors is metabolic dysregulation (MetD), defined here collectively as biomarkers including and associated with metabolic syndrome, insulin resistance, and visceral adiposity. Breast cancer survivors represent a distinctive group who endure many treatment-associated alterations in lifestyle habits including weight gain (8), reduced physical activity levels (9), and worsening metabolic profiles leading to metabolic dysfunction (10). Due to heightened adipose and systemic inflammation, and increased cell proliferation, MetD may increase the risk for cancer recurrence and mortality (11–13). These risks are even more pronounced in Latina and Hispanic populations, as MetD is 1.5 times more likely present in Latinas and Hispanics when compared to non-Hispanic white and African American women (14). Further, Latinas and Hispanics are less likely to participate in exercise, seek preventive medical attention or receive timely diagnosis, influencing the onset and severity of comorbidities, and negatively impacting survivorship (15).

An extensive body of evidence supports the benefits of exercise in breast cancer survivors to improve treatment-related side effects such as physical function, body composition, quality of life, musculoskeletal symptoms or fatigue (16–18). However, Latina and Hispanic breast cancer survivors (LHBCS) are often underrepresented in exercise interventions, with only three studies involving these populations (16, 19, 20). We previously reported the benefits of high intensity interval training on cardiorespiratory fitness among breast cancer survivors (73% Latina or Hispanic) receiving anthracycline chemotherapy (21). Additionally, we reported the benefits of a 16-week supervised aerobic and resistance exercise intervention among breast cancer survivors (57% Latina or Hispanic) and found that ethnicity modified the effects of exercise on cardiorespiratory fitness (VO_2max)_, physical and emotional well-being, metabolic syndrome, and sleep quality (22–24). Given these findings, there is a pressing need for culturally tailored exercise interventions (e.g., bilingual study materials and staff, culturally sensitive educational and program components, and accessible delivery) among LHBCS to better understand how exercise can attenuate health disparities, improve health outcomes, and reduce disease burden beyond treatment (19).

**Figure 1:**
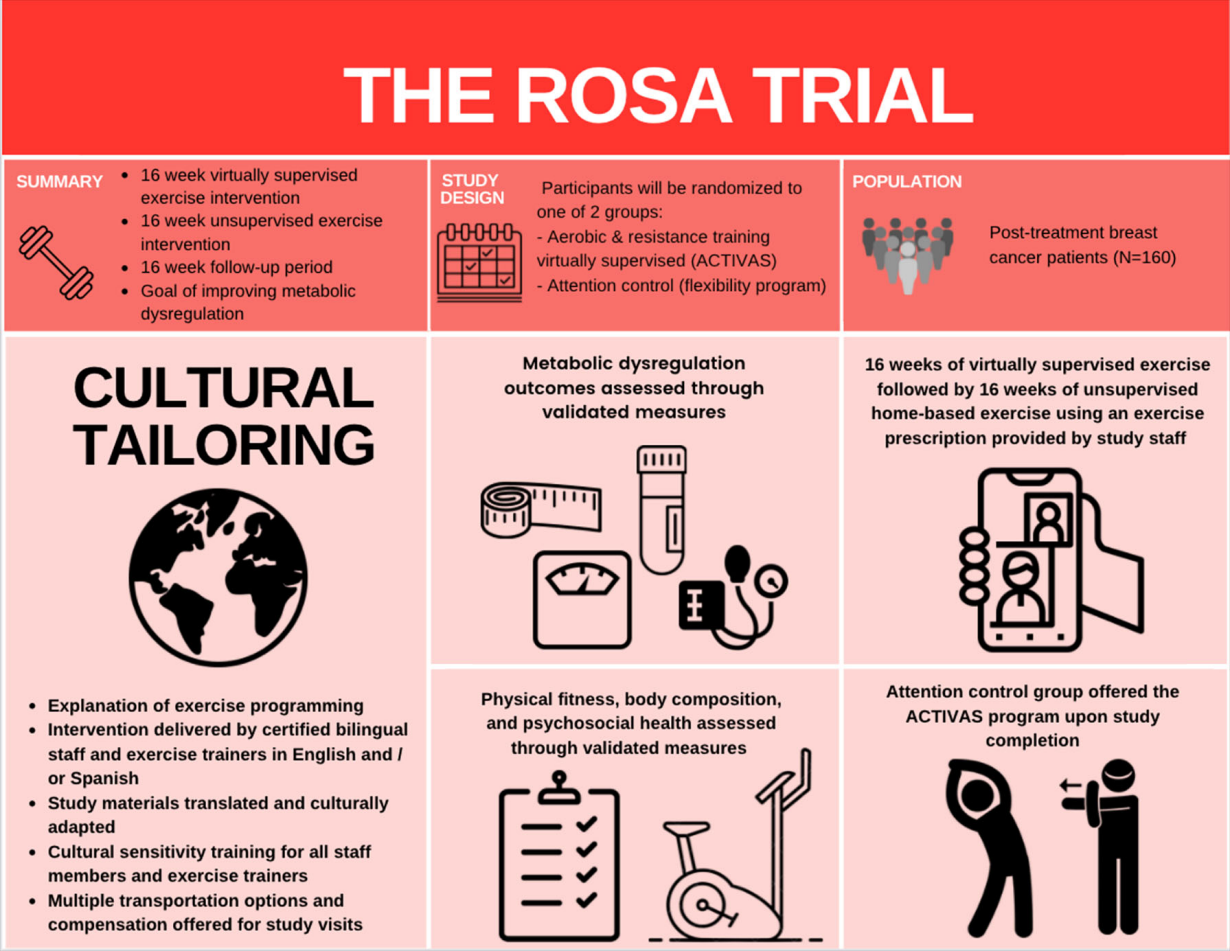
Conceptual Framework of the ROSA Trial.

Beyond the lack of representative exercise interventions, rates of participation in clinical cancer trials are low among disadvantaged and racial/ethnic minority groups (19, 25, 26), limiting the generalizability of results. It is imperative to bridge this research gap to address health disparities among the increasing number of LHBCS who experience poorer disease outcomes. Thus, we are currently conducting the ROSA trial, a randomized controlled trial to examine the effects of exercise on MetD in sedentary, obese LHBCS. We hypothesize that a 16-week supervised progressive aerobic and resistance exercise training program, followed by a 16-week unsupervised program will elicit improvements in MetD compared with the control group, and said improvements will be maintained among LHBCS during the 16-week follow-up (**Figure 1**).

## 2 Materials and Methods

### 2.1 Study Design

**Figure 2:**
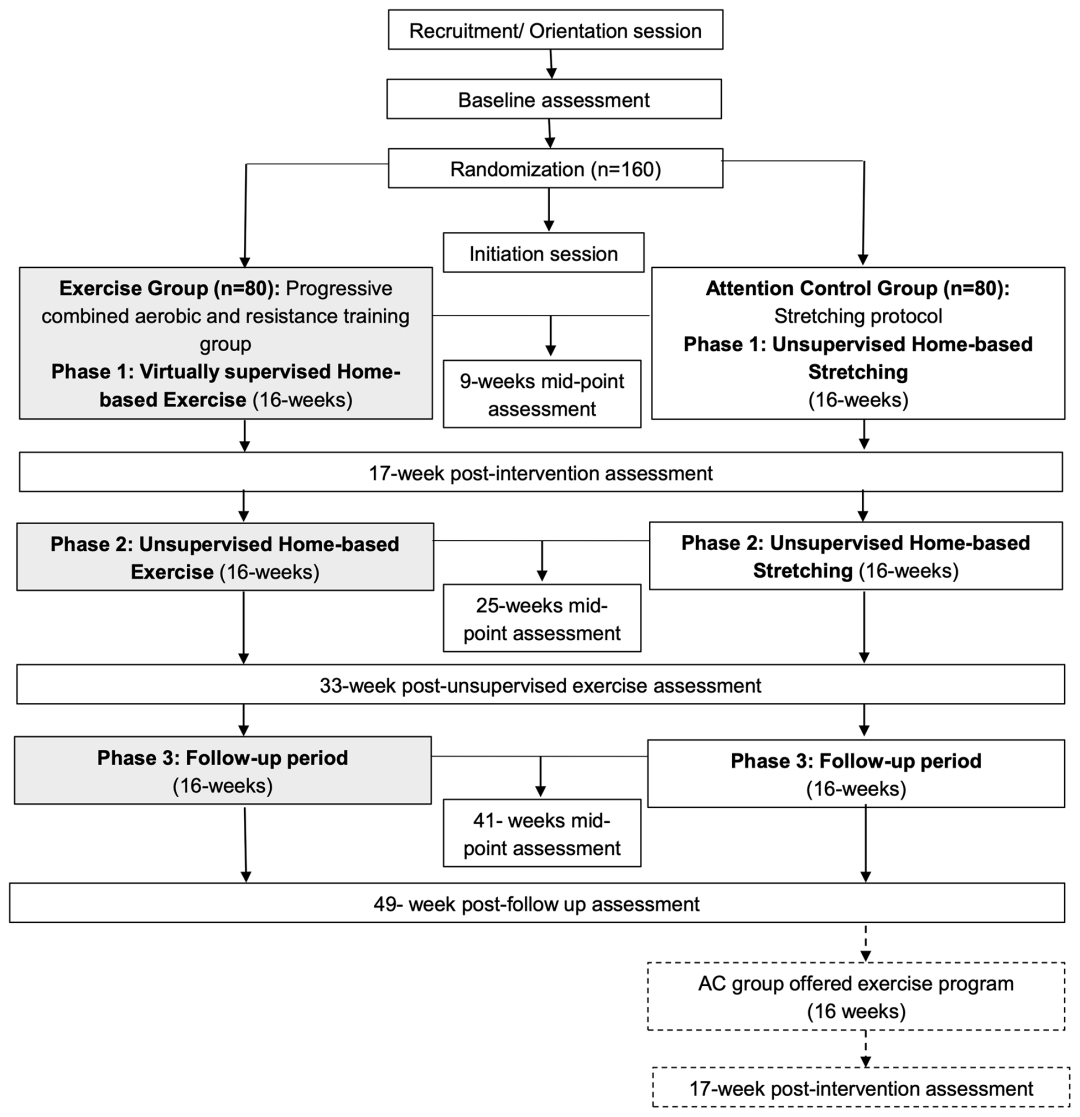
Study schema of the ROSA Trial.

**Figure 3:**
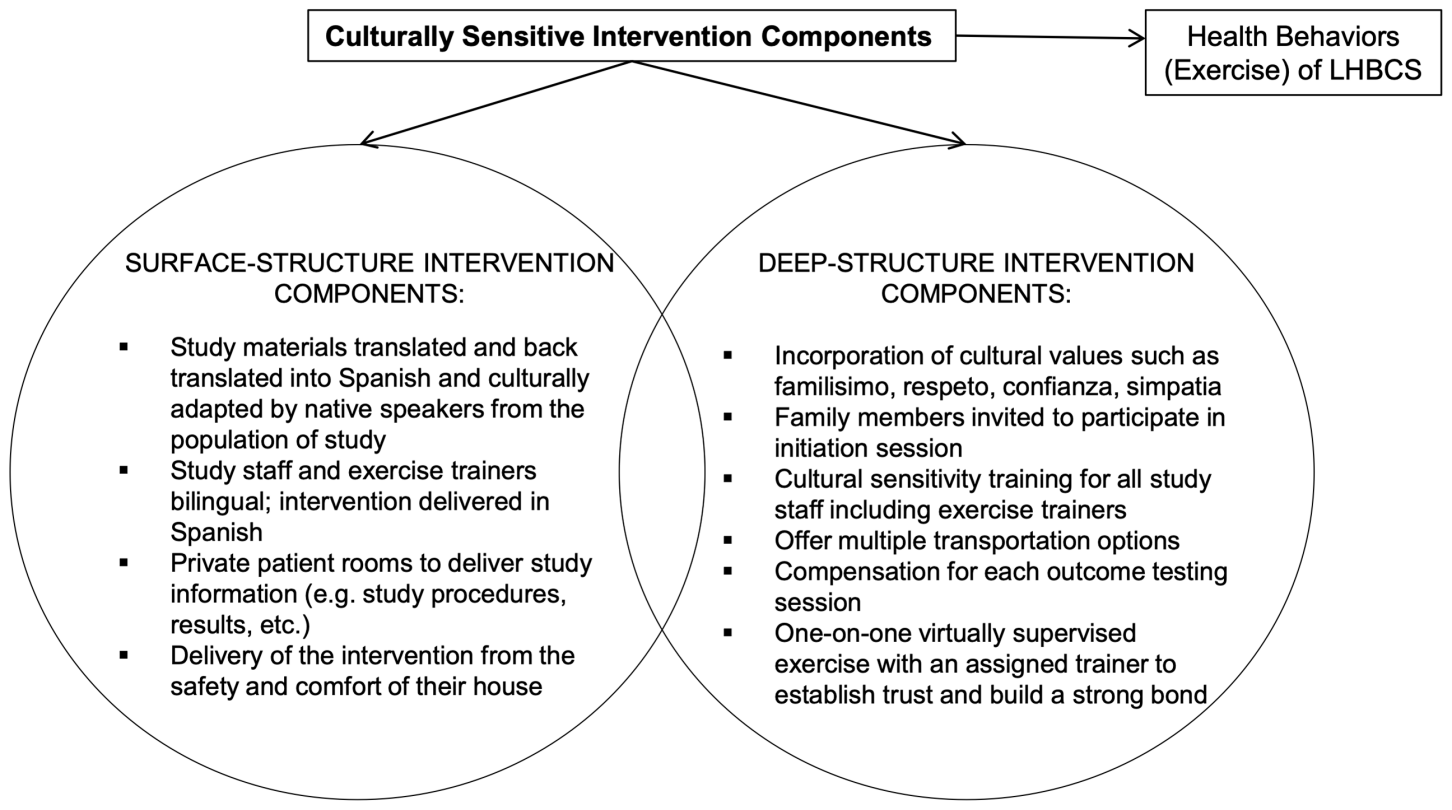
Culturally sensitive components of the ROSA Trial.

The ROSA trial is a 12-month single-center, two-armed, randomized controlled trial (RCT) underway at Dana-Farber Cancer Institute (DFCI), Boston, Massachusetts. A total of 160 centrally obese Latina and/or Hispanic women diagnosed with breast cancer who have completed cancer-related therapy (e.g., surgery, chemotherapy, and/or radiation) are recruited and randomly assigned to one of two groups (**Figure 2**): **A**erobic and resistan**C**e **T**rain**I**ng **V**irtu**A**lly **S**upervised (ACTIVAS) or attention control (AC). The ACTIVAS group participates in a three-phase design: phase 1 = 16-week virtually-supervised intervention, phase 2 = 16-week home-based unsupervised intervention, and phase 3 = 16-week self-directed exercise/follow-up period. The AC group receives a 12-month home-based stretching program. After the end of the intervention (12 months), the AC group will be offered the ACTIVAS program and will complete an additional testing session following the completion of the exercise intervention if elected. Various aspects of cultural tailoring are integrated throughout the ROSA trial described below (**Figure 3**).

### 2.2 Eligibility Criteria

To be eligible to participate in this study, women must meet the following inclusion criteria: 1) are ≥ 18 years of age with stage I-III breast cancer, 2) self-identify as Latina and/or Hispanic, 3) have undergone a lumpectomy or mastectomy, 4) have completed (neo)adjuvant chemotherapy and/or radiation therapy if prescribed; there is a required eligibility window of six weeks post-treatment to allow for recovery (participants may use adjuvant endocrine therapy if use will be continued for duration of study period), 5) clinical confirmation of no detectable cancer, 6) BMI >25 kg/m^2^ 7) have not experienced a weight reduction ≥10% within the past six months, 8) currently participates in less than 60 minutes of structured exercise per week, 9) non-smoker (no smoking during previous 12 months), and 10) willing to travel to DFCI, 11) signed informed consent. Exclusion criteria includes: 1) metastatic disease, 2) uncontrolled illness including active infection, diabetes, hypertension, or thyroid disease (27), 3) history of musculoskeletal, cardiorespiratory, or neurological diseases that preclude the participation in moderate intensity exercise, and 4) plans to undertake reconstructive surgery with flap repair during study period.

### 2.3 Recruitment and Screening

Participants are currently being recruited through various recruitment strategies at the DFCI and neighboring satellite clinics, including Partners Rally Platform, screening breast clinic lists, mass mail out through the Massachusetts Department of Public Health, and advertisements in patient centered newsletters and waiting rooms. For potential participants identified through patient lists, we contact providers, and request permission to contact the patients to invite them to participate in the study. To assess eligibility, all participants are screened *via* phone which includes a short questionnaire to determine eligibility and completion of the Godin Leisure Time Questionnaire (28) to assist in determining the participant’s current exercise level. To confirm the participant’s health status and how this relates to undertaking an exercise program we use the Physical Activity Readiness Questionnaire (29). To successfully recruit and retain LHBCS for participation in a clinical trial, we devised and pilot-tested a plan of action to ensure the feasibility of recruiting this population. We employed several strategies with all participants, including follow-up phone calls/emails after every visit, informative study brochures for recruitment and for each phase, Spanish-speaking research staff, flexibility in offerings of data collection and exercise training appointments, and transportation support resources (e.g. parking voucher, reimbursement for travel expenses, etc.). Approval of the trial protocol was obtained from the DFCI Institutional Review Board (IRB#20-221). Any amendments made will be communicated to all appropriate authorities. This clinical trial is registered in ClinicalTrials.gov (NCT04717050).

### 2.4 Orientation Session and Informed Consent

Prior to study enrollment and obtaining outcome measures, all participants are asked to attend one ~90-minute orientation session virtually, by phone, or in person led by the principal investigator, exercise training staff, and promotoras. During this session, study staff inform the participants and their families of the study details including the time commitment required, the study timeline, and tests performed. Past participants are invited to speak about their experiences in a clinical exercise program. Attending participants receive the informed consent form to review with study personnel in a private room (in person, virtually, or by phone) to clarify any remaining questions. Following the session, ample time is provided to allow participants to ask questions and decide on their participation before gaining written informed consent. Women who consented virtually or by phone are also asked to attend the session to obtain pertinent information for the study. Additionally, the oncology provider of each participant must also provide written consent confirming the health status and ability of the participant to partake in the exercise-based study.

### 2.5 Randomization and Blinding

After completing all baseline assessments, participants are randomly assigned to either the ACTIVAS exercise group or AC group using a 1:1 ratio and permuted blocked design with varying block sizes to ensure equal numbers of participants allocated to each group where investigators are blinded to this process. Our study biostatistician and co-investigator (H. U.) generated the randomization sheet as a cvs file prior to study start-up, after which the research coordinator M.N. uploaded the cvs file to a web-based application (REDcap). After baseline testing, one of our research assistants accesses REDCap to conduct randomization. Subsequently, our research assistant informs M.N. of randomization result and verbally informs the participant of their group allocation. Randomization is stratified by menopausal status (premenopausal and postmenopausal), which is evaluated at time of diagnosis. Testers and participants are blinded to group allocation during baseline testing, however, due to the nature of the study, it is not possible to blind the investigators or participants to group allocation after baseline. However, testers follow a detailed protocol and are trained in the importance of standardizing outcome assessments and avoiding bias.

### 2.6 Intervention

#### 2.6.1 Initiation Sessions

Following consent and obtaining outcome measures, participants in both groups attend an initiation session specific to the next phase (e.g., pre-Phase 1, pre-Phase 2, pre-Phase 3), during which the principal investigator, with support from the promotoras (community members with specialized training to provide basic health education) and exercise trainers, conduct one-on-one interviews following the Social Cognitive Theory (SCT) which is commonly used to guide exercise interventions for breast cancer survivors (30, 31), and recently noted as the most frequently utilized theory in culturally sensitive nutrition and exercise interventions for Latinx (32). The SCT construct emphasizes behavioral strategies that include: solving barriers to participate in exercise, instructions on how to monitor exercise using exercise log recall on a paper form and with an accelerometer, reducing barriers to access exercise equipment, discussion of the benefits of exercise with assessment of their perceived value of the benefits, and goal setting for each phase. An exit interview to revisit these strategies will be conducted at the end of the 12-month study period.

#### 2.6.2 Incorporation of Cultural Components

##### 2.6.2.1 Delivery of a Culturally Sensitive Intervention

Our intervention incorporates cultural considerations needed to tailor program delivery for LHBCS to offset barriers to participation in clinical trials and exercise, and to ensure a successful exercise intervention (e.g., explanations of exercise programming, exercise instructions by trainers, translated accompanying material describing the overall exercise program/clinical trial) (33). Thus, our program uniquely addresses cultural values and barriers to exercise among LHBCS (**Figure 3**). In summary, surface-structure components incorporated into the design include study materials in Spanish, Spanish-speaking study staff, recruitment by promotoras, and delivery of the clinical intervention from the safety and comfort of their own homes. At the deep-structure level, we focus on incorporating Latinxs cultural values to create a more culturally sensitive program. Specifically, to integrate the values of familisimo (strong identification with and attachment to nuclear and extended families), respeto (respect), confianza (trust), and simpatía (warmth, friendliness) (34), the study design invites family members to participate in the initiation/orientation session, offers multiple transportation options, includes compensation for each outcome testing session, conducts cultural sensitivity training for staff, and provides one-on-one virtually supervised exercise sessions with an assigned trainer to establish trust and build a strong bond.

##### 2.6.2.2 Exercise Training Personnel Workshops

All exercise training staff undergo a stringent exercise training educational program to ensure treatment fidelity and a cultural sensitivity workshop before initiating delivery of the intervention. The exercise training educational program is led by the principal investigator and conducted over two 4-hour sessions. Content includes a review of exercise techniques, exercise prescription, program goals, standardized verbal motivation and feedback, correctional cues, and safety regulations.

#### 2.6.3 Exercise Intervention (ACTIVAS)

For participants randomized to the ACTIVAS group, the intervention is divided into three phases:

##### 2.6.3.1 Phase 1 (Virtually Supervised Home-Based)

Participants in the ACTIVAS group participate in home-based, virtually supervised combined aerobic and resistance exercise sessions three times a week for 16 weeks. Each exercise session is supervised *via* Zoom by a certified cancer exercise trainer who has been formally trained by the principal investigator on the exercise program to ensure study specificity and adherence. Exercise equipment (stationary cycle, dumbbells and Fitbit) is provided and shipped to the participants’ houses after randomization, along with Wi-Fi-enabled tablets if participants do not have access to an appropriate streaming device.

The exercise intervention will start once the participant receives exercise equipment in their houses (~1 week after baseline testing). Exercise sessions *via* Zoom will start with a five-minute aerobic-based warm up. Then, the resistance exercise program will be performed prior to the aerobic training portion. The resistance program is conducted using dumbbells or resistance bands, depending on abilities, and consists of three sets of 10-15 repetitions of six different resistance exercises targeting all major muscle groups at 60-75% estimated one-repetition maximum (1RM), measured at baseline testing. Exercises include chair squat, floor chest press, glute bridge, seated bent over row, lunges, and shoulder press, however, exercises may be modified to accommodate changes in participant’s health and physical abilities.

Immediately following the resistance program, aerobic exercise is performed on the stationary cycle. Exercise intensity is prescribed using maximal heart rate achieved at peak oxygen uptake (VO_2peak_) as assessed by maximal cardiopulmonary exercise test (CPET) during baseline testing. Intensity begins at 50% of maximal heart rate and progresses to 85% by week 16, with duration ranging from 20-30 minutes over the 16-week period. Heart rate is monitored throughout the aerobic exercise sessions, using a Fitbit® heart rate monitor, to maintain the appropriate exercise intensity. Each exercise session culminates with five minutes of stretching and two core exercises.

Due to the progressive nature of the ACTIVAS intervention, VO_2peak_ and muscle strength (estimated 1-RM values) are tested again at week nine to allow for accurate adjustments of resistance and aerobic intensities as the participants progress over the exercise intervention. **Table 1** displays a sample of the exercise program parameters at week 1-16, showing the progression of exercise.

**Table 1:** Overview of Phase 1: Virtually supervised home-based intervention (ACTIVAS program).

Weeks 1-16: Virtually supervised Home-Based ACTIVAS Program							
	Resistance Exercise			Aerobic Exercise		Core Exercise	
Mesocycle	Volume (Sets)	Volume (Reps)	Intensity (1RM)	Intensity (VO2_Peak_→HRmax)	Volume	Type	Volume
**Mesocycle 1**							
Week 1	3	10	60%	50%	20min 3x/wk	1) High plank (couch/wall/chair)	3x30s
Week 2	3	10	60%	50%	20min 3x/wk	2) Crunches	3x15
Week 3	3	15	65%	55%	25min 3x/wk	1) Alternate arm/leg extension	3x15ea
Week 4	3	15	65%	55%	25min 3x/wk	2) Reverse crunch	3x15
**Mesocycle 2**							
Week 5	3	15	70%	60%	30min 3x/wk	1) High plank (floor)	3x30s
Week 6	3	15	70%	60%	30min 3x/wk	2) Side crunches	3x10ea
Week 7	3	15	75%	65%	30min 3x/wk	1) Low plank (couch)	3x30s
Week 8: De-Load/Test	3	10	65%	65%	20min 3x/wk	2) Bicycle crunches	3x10ea
**Mesocycle 3**							
Week 9	3	15	60%	70%	30min 3x/wk	1) Low plank (floor)	3x30s
Week 10	3	15	60%	70%	30min 3x/wk	2) Alternate leg lifts	3x10ea
Week 11	3	15	65%	75%	30min 3x/wk	1) High plank with knee drive	3x10ea
Week 12	3	15	65%	75%	30min 3x/wk	2) Reverse crunches	3x15
**Mesocycle 4**							
Week 13	3	15	70%	80%	30min 3x/wk	1) High plank with shoulder tap	3x10ea
Week 14	3	15	70%	80%	30min 3x/wk	2) Sit-up (arms crossed)	3x15
Week 15	3	15	75%	85%	30min 3x/wk	1) High plank with leg lift	3x10ea
Week 16	3	15	75%	85%	30min 3x/wk	2) Sit-up (hands behind head)	3x15

Ea, each; HR, heart rate; Min, minutes; Reps, repetitions; S, seconds; VO2_peak_, peak oxygen uptake; 3x/wk, 3 days per week.

##### 2.6.3.2 Phase 2 (Unsupervised Home-Based)

During phase 2, the ACTIVAS group receives an individualized 16-week, thrice weekly, home-based exercise program provided in the form of a handbook, which is not virtually or actively supervised by study staff. The program consists of three combined resistance and aerobic exercise sessions per week. The resistance exercise program consists of six exercises targeting all major muscle groups completed at an intensity of 60-75% estimated 1RM for three sets and 10-15 repetitions. The aerobic exercise program consists of stationary cycling progressing from 60 to 85% VO_2peak_/heart rate maximum (calculated from the CPET) at varying durations ranging from 20 to 30 minutes. The assigned trainer follows up with each participant on a weekly basis by phone call, email, or text message to assess program adherence, answer any questions, and document barriers to exercise. If the participant does not answer, a message is left. If the participant is unresponsive for over a week, the exercise sessions are recorded as missed unless trainer is contacted by the participant and an update is given. Participants are asked to record any form of daily structured exercise including type, duration, and intensity in a physical activity log booklet. Due to the progressive nature of the ACTIVAS intervention, VO_2peak_ and muscle strength (estimated 1-RM values) will be tested at week 25 to further progress the aerobic and resistance training. **Table 2** displays an overview of the exercise program at weeks 17-32.

**Table 2:** Overview of Phase 2: Unsupervised home-based intervention (ACTIVAS program).

Weeks 17-32: Unsupervised Home-Based ACTIVAS Program							
	Resistance Exercise			Aerobic Exercise		Core Exercise	
Mesocycle	Sets	Reps	Intensity (1-RM)	Intensity (VO2_Peak→_HRmax)	Volume	Two core exercises are selected by the participant per session and include:	
**Mesocycle 1**						High plank Low plank Alternate arm/leg extension Crunches Side crunches Bicycle crunches Alternate leg lifts Knee drive plank Reverse crunch Shoulder tap plank Sit-up Plank with leg lift	3x30s 3x30s 3x15ea 3x15 3x15ea 3x15ea 3x15ea 3x15ea 3x15 3x15ea 3x15 3x15ea
Week 17	3	10	60%	60%	30min 3x/wk		
Week 18	3	10	60%	60%	30min 3x/wk		
Week 19	3	15	65%	65%	30min 3x/wk		
Week 20	3	15	65%	65%	30min 3x/wk		
**Mesocycle 2**							
Week 21	3	15	70%	70%	30min 3x/wk		
Week 22	3	15	70%	70%	30min 3x/wk		
Week 23	3	15	75%	75%	30min 3x/wk		
Week 24: De-Load/Test	3	10	65%	65%	20min 3x/wk		
**Mesocycle 3**							
Week 25	3	15	60%	70%	30min 3x/wk		
Week 26	3	15	60%	70%	30min 3x/wk		
Week 27	3	15	65%	75%	30min 3x/wk		
Week 28	3	15	65%	75%	30min 3x/wk		
**Mesocycle 4**							
Week 29	3	15	70%	85%	30min 3x/wk		
Week 30	3	15	70%	85%	30min 3x/wk		
Week 31	3	15	75%	85%	30min 3x/wk		
Week 32	3	15	75%	85%	30min 3x/wk		

Ea, each; HR, heart rate; Min, minutes; Reps, repetitions; S, seconds; VO2_peak_, peak oxygen uptake; 3x/wk, 3 days per week.

##### 2.6.3.3 Phase 3 (Follow-Up Period)

Participants in the ACTIVAS group are encouraged to participate in self-directed exercise with no communication or exercise prescription from study staff for the 16-week duration of Phase 3. During this time, participants continue to log their activity as per phase 2.

#### 2.6.4 Attention Control Group (AC)

This group performs a home-based program of the same stretches utilized in the ACTIVAS group. The stretching protocol consists of one set of 3-4 static stretching exercises held for 30 seconds and performed three days per week. As flexibility exercises are low-intensity, low-impact and low-volume, minimal caloric expenditure is expected to be incurred. To increase compliance and aid in the standardization of the home-based stretching, participants are provided a booklet of the flexibility exercises (35, 36). Participants are shown how to use the booklet and instructed in the stretching exercises by an exercise trainer prior to the intervention. In addition, participants are asked to complete weekly records of flexibility compliance and physical activity performed outside the study. The AC group is followed throughout the 12-month study duration and are completing activity logs during this period. At 12 months, the AC group are invited to participate in Phase 1 of the ACTIVAS intervention. If elected, participants attend one additional testing visit after the 16-week exercise intervention at week 65. The assigned trainer follows up with each participant on a weekly basis by phone call, email, or text message to assess program adherence, answer any questions, and document barriers to continue in the study.

#### 2.6.5 Intervention Adherence

If exercise sessions are missed, make-up sessions are scheduled as soon as possible with no more than four supervised sessions allowed per week. All participants are given an additional two weeks beyond the 16-week supervised intervention period to make up for any exercise sessions missed due to illness, work, travel etc. Participant adherence to the ACTIVAS program will be captured by, 1) percentage and number of prescribed sessions attended (participants must attend ≥80% of sessions to be considered compliant with ACTIVAS program e.g., ≥38 sessions), and 2) average minutes of exercise/week (participant must complete ≥80% of prescribed minutes) performed at the prescribed intensity.

#### 2.6.6 Adverse Events

Any expected and unexpected adverse events are reported to the principal investigator who then subsequently reports to the institutional review board. Serious events must be reported within 24 hours of occurrence or finding out about the event. All adverse events, both serious and non-serious, and deaths that are encountered from initiation of study intervention, throughout the study, and within 30 days of the last study intervention are followed to their resolution, or until the participating investigator assesses them as stable or determines the event to be irreversible, or the participant is lost to follow-up. The presence and resolution of adverse events are documented on the appropriate case report form and recorded in the participant’s medical record. Furthermore, given the remote nature of the exercise sessions, additional safety precautions include: 1) providing safety recommendations specific to exercises and/or assessments (i.e., standing near a wall for balance activities), 2) adapting the exercise protocol as necessary (i.e. limit weight, changing resistance exercise modality), 3) verifying address of remote exercise location (i.e., home address), should study staff need to call 911 in an emergency, and 4) ensuring that emergency contact information of the participant is up-to-date and readily available.

### 2.7 Outcome Measures

**Figure 4:**
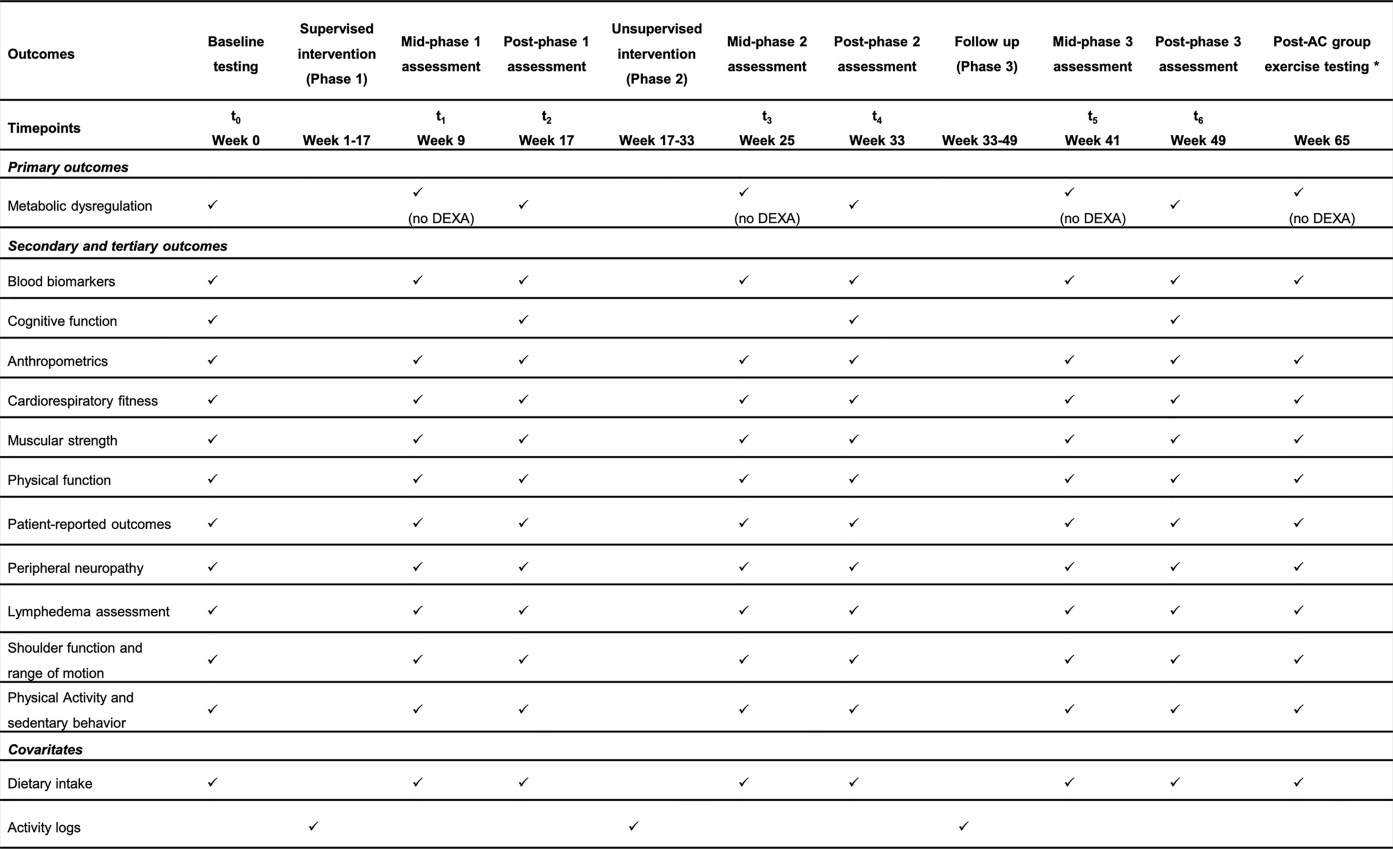
SPIRIT flow diagram for study visits timeline of the ROSA Trial. DEXA, dual-energy x-ray absorptiometry; AC, attention control group. *Only for the AC who choose to complete the ACTIVAS program.

Testing is completed across two days at time points baseline (Week 0), post-phase 1 (Week 17), post-phase 2 (Week 33), and post-phase 3 (Week 49), with only one day required for testing at all mid-point assessments (Week 9, 25, 41). A complete data collection schedule is provided in **Figure 4**. All measures are collected at all time points and both groups unless specified below. Participants will be provided with monetary compensation (25$) for each testing timepoint attended, and parking validation for every visit to DFCI. All measures that have a verified Spanish version will be used for those who are Spanish speaking and/or would prefer to complete testing in Spanish.

#### 2.7.1 Primary Outcomes

Metabolic dysregulation is assessed as (a) insulin resistance (primary endpoint), (b) visceral adiposity, and (c) frequency of metabolic syndrome.

Homeostasis Model Assessment (HOMA) is used to estimate insulin resistance using fasting plasma levels of glucose and insulin (37). Due to cost and the invasive nature of directly quantifying insulin resistance (e.g. glucose clamp), we chose to use HOMA as a surrogate measure of insulin resistance, which is a validated measure in clinical studies (38).

A whole-body dual-energy x-ray absorptiometry (DEXA) (*Hologic Inc., Marlborough, MA*) scan is used to assess visceral adiposity, in addition to measures of whole body and appendicular and whole-body fat and lean mass (kg). Although central obesity is generally defined as a waist circumference >80 cm in women (39), capturing an image of visceral adiposity tissue provides a more accurate portrayal of central obesity. DEXA is strongly correlated with computed tomography (40) and is a practical and quick method of assessing visceral adiposity among breast cancer survivors. Automated visceral adiposity analysis is used to quantify the volume (cm^3^) of visceral adiposity tissue in the abdomen. DEXA is only to be performed at testing visits during baseline, post-phase 1, post-phase 2 and post-phase 3, and will not be performed during mid-phase assessments (**Figure 4**).

Frequency of metabolic syndrome is captured based on the metabolic syndrome criteria accepted by the American Heart Association (AHA), consisting of hypertension, high waist circumference, and elevated levels of serum HDL-cholesterol, triglycerides, and fasting glucose (39):

- Blood pressure (BP) is measured using an automated device with an appropriately sized cuff (*Omron BP 786, Lake Forest, IL*) after the participant has sat quietly for five minutes while resting their arm on a table so the brachial artery is level with the heart.
- Waist circumference (cm) defined as the distance around the waist using the umbilicus as the reference point and is assessed with a constant-tension tape measure.
- Lipid profile and fasting glucose are obtained from a fasting (12 hour fast) blood draw by a DFCI nurse or phlebotomist. After being treated and aliquoted, the blood serum and plasma samples are stored in a -80°C freezer for future batch-tested analysis. Analysis of bloods through commercially available ELISA kits (*ThermoFisher Scientific, Waltham, MA*) will be used. A multitude of cancer-related biomarkers will be examined including but not limited to leptin, IL-6, TNF-a, adiponectin, estradiol, and SHBG.

#### 2.7.2 Secondary Outcomes

##### 2.7.2.1 Anthropometrics

Height (cm) is assessed using a stadiometer with shoes removed. Hip circumference (cm), defined as the distance around the widest girth of the buttocks using the greater trochanter as a landmark, is measured with a constant tension tape measure. Weight (kg) and body composition is assessed using bioelectrical impedance (*Tanita 780, Arlington Heights, IL*), with shoes and socks removed, providing both appendicular and whole-body measures of fat and lean mass (kg). Height and weight are used to calculate body mass index (BMI, kg/m^2^).

##### 2.7.2.2 Cardiopulmonary Fitness and Exercise Capacity

To assess VO_2peak_ a maximal CPET is completed on a cycle ergometer (*ErgoSelect 100, Ergoline, Germany*) using an incremental ramp protocol (41, 42). Participants complete a 5-minute warm-up at a resistance of 40W then proceed into an incremental ramp protocol increasing 10W every minute until volitional fatigue. Cadence is maintained between 60 and 80 revolutions per minute (41, 42). Heart rate (*Polar USA, Lake Success, NY*) and rate of perceived exertion (RPE; Borg scale 1-10) are recorded every minute and at cessation of test. Expired gas analysis (*TrueOne 2400, ParvoMedic Inc., Salt Lake City, UT*) is used to measure VO_2peak_. The results of this test are also used to calculate the target heart rate to achieve the prescribed exercise intensity during the aerobic program.

The 6-minute walk test is used to estimate exercise capacity (43, 44). Participants are instructed to walk as far as they can in 6-minutes walking up and back a 10m walking course within a hallway. Distance achieved in six minutes is measured in meters.

##### 2.7.2.3 Muscular Strength

Isometric handgrip strength is measured in the dominant upper limb (*Camry Digital Hand Dynamometer, El Monte, CA*). Participants perform three trials in a standing position with their arms down by their side with the highest result used for analysis.

Muscular strength is assessed by estimated 1RM calculated from 10RM of leg press and chest press (*Matrix Fitness, Cottage Grove, WI*). Additionally, 10RM is completed for each of the six resistance intervention exercises prescribed so that an accurate intensity may be prescribed: 1) chair squat, 2) floor chest press, 3) glute bridge, 4) seated bent over row, 5) lunges, and 6) shoulder press. Participants perform 1-2 warm-up sets of 6-8 repetitions. The participant proceeds to complete sets of 10 repetitions with increasing weight each set until volitional fatigue is reached on the 10^th^ repetition. The weight of the participant’s 10RM is recorded in kilograms and used to estimate 1RM using validated equations (45, 46).

##### 2.7.2.4 Physical Function

Physical function is assessed using the short physical performance battery (SPPB) (47), timed up and go (TUG) (48), gait speed (49), Margaria stair climb (50), and sit-to-stand (51) tests. The SPPB is comprised of three sections: 1) balance with feet together, semi tandem, and full tandem is held for up to 10 seconds (s) with no support. Only one attempt is given for each position, and the time (s) to complete is recorded; 2) usual gait speed over 4m is timed where the participant completes two attempts with the fastest time (s) recorded; and 3) chair stand where the time (s) to complete five chair sit-to-stands is recorded, only one attempt is given, after a familiarization practice. Each section of the SBBP is given a score dictated by performance to then provide a summary score. The TUG test times how fast it takes a participant to stand up from a chair, walk around a cone placed 3m away from a chair where they start, and end in a seated position. Participants attempt the TUG three times, after a familiarization practice, where the average of the three trials is calculated. Gait speed is assessed over a 6m flat surface where the time to walk the 6m course at a usual and fast pace is recorded. Two attempts for each speed are completed. The Margaria stair climb is completed in a stairwell of ten stairs. The participant walks/runs up the stairs as fast, and safely, as they can. The time (s) the participant takes from stair three to stair nine is recorded; three attempts are given after a familiarization practice, where the average time of the three trials is calculated. Power (kg) is calculated from the stair climb using a validated equation (50). The sit-to-stand test involves participants completing as many sit-to-stands from a seated chair position to a standing position with full hip extension in 30 s; one attempt is given, with number of full movements with correct technique recorded.

##### 2.7.2.5 Lymphedema

Lymphedema has been defined in recent literature as a greater than 10% difference in volume calculation for the arm compared to the uninvolved upper extremity (52). Lymphedema is assessed using geometric arm volume calculations on both upper limbs (52). Circumferential measurements are taken with a constant-tension tape measure at the following anatomic landmarks: axillary fold, halfway between axillary fold and antecubital fossa, antecubital fossa, halfway between antecubital fossa and wrist, and wrist. Calculations for limb geometric volume are performed using the frustum (truncated cone) volume as described by Taylor et al. (52). A percentage difference between lymphedema limb and the uninvolved limb is calculated to determine the amount of lymphedema.

##### 2.7.2.6 Shoulder Function and Range of Motion

Upper body function is assessed using the Y Balance Test Kit (*Move2Perform, Evansville, IN*) (53). Participants begin in a three-point plank position on toes or knees depending on ability. The tested shoulder is the supporting limb placed on the connecting block, the non-testing limb is placed on the medial block, and feet are placed shoulder-width apart. From this position, the participant uses their non-testing limb to move each reach block one at a time in the medial, inferolateral, and superolateral directions in a controlled manner as far as they can and then return to the starting position. The test is performed three times on each side with 30s rest between trials. The reach distance of each direction is recorded where the highest value of each direction is used to calculate an average composite score.

Bilateral upper body strength and power is assessed through the seated medicine ball throw (SMBT) (54). Participants are instructed to sit on the floor with their head, shoulders, and back against the wall and legs extended; legs may be bent if ability does not allow for full extension. To calculate a relative throwing distance, arm reach distance is measured from the wall in meters. Using a two kg medicine ball covered in chalk, the participant performs a chest pass holding the ball with both hands and throwing it forward in a straight line as far as they can with head, shoulders, and back maintaining full contact with the wall. A measuring tape is used to measure the distance from the wall to the most proximal point of the chalk mark with the participant’s reach distance subtracted to provide a final distance in meters. Three test trials will take place after a practice throw with a 1-minute rest between each trial. The average of the three trials will be calculated.

Shoulder function is assessed through the shoulder performance test (SPT) using the following tasks: overhead reach, hand behind the head, and hand behind the back. All movements are completed in a standing position starting with arms by side. The time(s) taken to complete the respective tasks with the correct technique 20 times is recorded. Each task is repeated right and left.

Upper body function is assessed using the closed kinetic chain upper extremity stability test (CKCUEST) (55). Participants begin in a three-point plank position on toes or knees, depending on ability, with hands 36 in. (91.4 cm) apart, shoulders perpendicular to hands, and feet hip-width apart. From this position, the dominant hand reaches across the body, touches the non-dominant hand, and returns to the starting position. Subsequently, the same movement is performed by the non-dominant hand. Participants are instructed to perform as many alternating touches as possible in 15s while maintaining the correct push-up position. Three trials occur after practice with 45s of rest in between each trial.

Shoulder active range of motion (AROM) is measured on both upper limbs. Using a goniometer (*Jamar E-Z Read*), AROM is assessed in external rotation at 0°, external rotation at 90°, forward flexion, and abduction (56, 57). The participant stands for all tests except for external rotation at 90° where the participant is lying on the ground with knees bent and feet hip-width apart. The starting position of the measured limb is dictated by AROM being tested. The participant is asked to actively perform the required motion with the angle of movement recorded in degrees. Three active trials are performed after the tester passively moves the participant’s limb in the tested motion.

Upper limb musculoskeletal disorders are assessed using the Disabilities of the Arm, Shoulder, and Hand (DASH). DASH is a 30-item questionnaire designed to measure physical function and symptoms of possible musculoskeletal disorders of the upper limb (intraclass correlation coefficient = 0.96; 95% CI = 0.93-0.98) (58).

##### 2.7.2.7 Physical Activity and Sedentary Behavior

Physical activity and sedentary behavior are additionally assessed using the ActiGraph wGT3X-BT (*ActiGraph LLC, Pensacola, FL*) at all testing time points. Participants wear the accelerometer on their hip for seven consecutive days excluding water-based activities and sleep. ActiLife software (*ActiLife 6; ActiGraph LLC*) is used to analyze the ActiGraph data. Only wake wear time is used with a minimal data collection period set for inclusion in analysis of four days of at least 600 minutes per day. Non-wear time is excluded from the analysis, defined as ≥90 minutes of consecutive zeros with a 2-minute spike tolerance (59). Commonly used cutoff points among cancer patients will be used to classify sedentary time (<100 counts per minute), light physical activity (100–1951 counts per minute), and moderate-to-vigorous physical activity (≥1952 counts per minute) (60–62).

##### 2.7.2.8 Patient-Reported Outcomes

Health-related quality of life (QOL) is assessed using the Functional Assessment of Cancer Therapy-Breast (FACT-B) questionnaire. The FACT-B is comprised of 44 items to specifically assess QOL in breast cancer patients (internal consistency coefficient of α = 0.90) (63). The Brief Fatigue Inventory (BFI) is used to rapidly assess the severity and impact of cancer-related fatigue including six items that correlate with QOL measures (internal consistency coefficient of α = 0.96) (64). Depressive symptoms are assessed using the 20-item Center for Epidemiologic Studies Depression (CES-D) scale, which was designed to measure one’s current level of depressive state (internal consistency coefficient of α = 0.82) (65, 66). The State-Trait Anxiety Inventory (STAI) is a 40-item questionnaire, which will be used to examine anxiety, with a consistency coefficient of α = 0.86-0.95 (67). Sleep quality is assessed using the Pittsburg Sleep Quality Index (PSQI) which contains 19 questions evaluating seven domains of sleep: subjective sleep quality, sleep latency, sleep duration, habitual sleep efficiency, sleep disturbances, use of sleep medication, and daytime dysfunction (internal consistency coefficient of α = 0.83) (68, 69). The Brief Pain Inventory – short form (BPI) is used to assess the impact of pain on QOL. The BPI includes a sensory and reactive dimension and has been previously validated in breast cancer survivors (internal consistency coefficient of α = 0.81-0.89) (70, 71). Barriers to recruitment and exercise adherence are assessed using the 17-item Barriers to Recruitment Participation Questionnaire (BRPQ) (72), and the 43-item Exercise Benefits/Barriers Scale (EBBS) (73). Participant burden is assessed using the 21-item, Perceived Research Burden Assessment (PRBA) (internal consistency coefficient of α = 0.87-0.96) (74).

##### 2.7.2.9 Cognitive Function

Cognitive function is only assessed at baseline, post-phase 1, post-phase 2, and post-phase 3 to prevent familiarization with the cognition-based questions. To assess executive functioning and abilities including response inhibition, cognitive flexibility, working memory, planning, insight, social cognition and behavior, and verbal fluency, we use the NIH toolbox (www.nihtoolbox.org) and the Montreal Cognitive Assessment to assess global cognition. Within the NIH toolbox the following tests are administered: Auditory Verbal Learning Test for immediate recall (memory), Picture Sequence Memory Test for episodic memory, Oral Reading Recognition for language, Flanker for executive function and attention, List Sorting Test for working memory, Oral Symbol Digit Test for processing speed, Dimensional Change Card Sort Test for executive function, and Pattern Comparison for processing speed. We also include an episodic memory composite that is derived from tests examining the hippocampus and surrounding medial temporal lobe (75).

##### 2.7.2.10 Peripheral Neuropathy

Peripheral neuropathy is measured through two tests: 1) Semmes-Weinstein Monofilament Examination (SWME), and 2) vibration testing by the on-off method (76–78). For both tests, participants are seated in an upright position with their eyes closed and socks and shoes off. The SWME is used to evaluate protective sensation at two sites: 1) pad of the great toe (foot), and 2) pad of the index finger (hand), where the nylon monofilament is applied four times to each site and the participant verbally identifies whether the left or right appendage is being touched. The vibration sensation is evaluated by applying a tuning fork to the bony prominences of the great toe, thumb, and medial malleolus where the participant verbally identifies the stopping of the vibration movement. Correct identification of side of the body and on/off vibration sensation for the respective tests is recorded.

#### 2.7.3 Covariates and Other Measures

##### 2.7.3.1 Sociodemographic, Health Behavioral, and Medical Profiles

Sociodemographic, health behavioral, and medical profiles are collected using a set of questions self-reported by the participants.

##### 2.7.3.2 Dietary Intake

Recent dietary patterns are assessed using an automated self-administered 24-hour dietary assessment tool (79). Participants complete three assessments at home on two weekdays and one weekend day, recording all food and drink consumed during the previous 24-hour period. Participants’ consumption of macro- and micronutrients will be analyzed.

##### 2.7.3.3 Activity Logs

The AC group completes a daily activity log for each week throughout the 12-month study period. The ACTIVAS group is asked about their physical activity completed outside of the supervised sessions over the previous week. Additionally, ACTIVAS group completes daily activity logs during the unsupervised and follow-up periods only. The participants are asked to record the type of exercise performed, time spent undertaking the exercise, intensity of that exercise bout (RPE 1-10 Borg scale, or average heart rate for the ACTIVAS group if they are wearing a heart rate monitor), and day of the week it was completed (frequency).

### 2.8 Data Monitoring and Management

Data is monitored internally within DFCI for timeliness of submission, completeness, and adherence to protocol requirements. Monitoring begins at the time of participant registration and continues during protocol performance and completion. The study team collects, manages, and performs quality checks on the data. Potential audits or inspections may be conducted by the principal investigator or their designated representatives. All data is stored on a secure network drive using REDCap, a HIPAA compliant web-based application hosted by Partners HealthCare Research Computing, Enterprise Research Infrastructure & Services, on password protected computers. Any hardcopy data is stored in locked filing cabinets in card access facilities. Results of this study will be presented in publication, conference, and invited speaker formats.

### 2.9 Sample Size Calculation

We propose to randomize 160 subjects (n=80/group). We assume 15% attrition (which is exceptionally conservative given our retention rates of >90%) (80) at the primary endpoint (4-months). With this sample size, we consider the study is sufficiently powered to detect a between-group difference. Specifically, the primary efficacy variable of this study is change in HOMA-IR from baseline to 4-month). From the preliminary results (80), we anticipate the standard deviation of HOMA-IR change from baseline is 6.9. The study will have 80% power, at a two-sided 0.05 alpha level, to detect a 3.3 difference in HOMA-IR change from baseline between the groups.

### 2.10 Statistical Analysis

The primary analysis population is all randomized subjects based on the intention-to-treat principle. The secondary analysis population is the per-protocol set. To test hypotheses related to differences in MetD outcomes across groups at the primary endpoint (4-month), we will use two-sample t-test for the continuous outcomes (HOMA-IR and VA) and Fisher’s exact test for the binary outcomes (diagnosis of metabolic syndrome). For HOMA-IR and VA, we will calculate change from baseline at 4-month for each subject and estimate mean difference between groups and corresponding 95% confidence interval. For diagnosis of metabolic syndrome, we will estimate the proportion of participants with metabolic syndrome at 4-month for each group and compare it between groups. Odds ratios and corresponding 95% confidence interval will be used to summarize the magnitude of the effect of the intervention. ANCOVA and logistic regression models will be also used to compare each of these outcomes at 4-month between groups, conditioning on potential prognostic factors, such as menopausal status, age, BMI, months since end of treatment, history of diabetes, type of treatment (chemotherapy vs radiation vs both), surgery type (lumpectomy, mastectomy), previous history of physical activity prior to diagnosis, and family medical history self-reported.

These primary endpoints are measured longitudinally. We will fit the longitudinal data with generalized mixed-effects models. The identical link and logistic link will be used for continuous and binary outcomes, respectively. The group indicator, time, and other covariates will be included in the models as the fixed effects. Some of the covariates (e.g., menopausal status) will be time-dependent covariates. Measures of physical fitness, functional capacity, and patient-reported QOL outcomes will be also analyzed in the same way.

Subgroup analyses will be conducted to assess the heterogeneity of the intervention effect on each outcome. The following factors will be considered: menopausal status, age, BMI, months since end of treatment, previous diabetes.

The primary analysis would include all completed outcome assessments (regardless of whether the woman stayed on the intervention), inviting those who drop from the intervention to return for outcome assessments. The only missing data here would be those who drop the intervention and do not return for outcome assessments; this includes participants withdrawn due to noncompliance. Preliminary analyses will compare women who do and do not contribute to this analysis on baseline characteristics. A sensitivity analysis will include multiple imputation of all missing outcome data for all time points. While the analytic methods specified for the longitudinal data are likelihood-based and provide valid results when missing mechanism is missing at random, it is possible that missing data may be informative and impact inference of the intervention effect on outcomes. Various sensitivity analyses for missing observations will be performed to assess the robustness of conclusions derived from the primary analysis.

## 3 Discussion

The ROSA trial is the first exercise oncology trial to focus on physically inactive LHBCS with obesity and comprehensively measured health outcomes. Specifically, this trial employs three phases of study period over 12 months: (1) we will determine the effects of 16-weeks virtually supervised progressive combined aerobic and resistance training on the primary outcome of MetD, a comprehensive health composite score (Phase 1); (2) Then, we provide a 16-weeks unsupervised home-based exercise program to support maintenance of exercise-induced health benefits from Phase 1 and to support exercise participation without direct supervision (Phase 2); and (3) Next, we examine whether exercise behaviors and changes in MetD outcomes can be maintained during a 16-week follow-up period when supervision and exercise programming is removed (Phase 3). Lastly, we will explore the effects of virtually supervised and unsupervised home-based exercise training on other important health markers such as physical fitness and function, cognition, and patient-reported outcomes.

The ROSA trial targets an ethnic minority population of LHBCS who have been largely marginalized in previous research (19). The ROSA trial was designed in part by the results obtained from our previous trial (80). We previously reported that LHBCS had poorer physical fitness, and were younger and more likely to have advanced stage cancer compared with non-LHBCS breast cancer survivors. Additionally, among the same sample, we reported that LHBCS improved their metabolic profile more than non-Latina breast cancer survivors after 16-week supervised aerobic and resistance training (23). Moreover, our results showed that following the exercise intervention, LHBCS were more likely to experience larger benefits from exercise on physical and emotional well-being, cardiorespiratory fitness, and sleep quality compared to non-Latina survivors (22, 24). In addition to our trial, we found two previous studies focusing on exercise interventions in this specific population. Alexis et al. (81) reported that a home-based resistance and aerobic program improved muscle strength and range of motion in LHBCS. Moreover, the Project VIVA, found that LHBCS increase their levels of physical activity with exercise interventions that involved social support from family and friends (82). Therefore, given these findings and the lack of clinical trials in this population, we sought to conduct an exercise oncology trial solely focused on this vulnerable, highly understudied population to address the research gaps within exercise oncology.

The lack of inclusion of Latina and Hispanic populations in oncology research in general may be due to several reasons. In cancer screening, prevention, and treatment trials, unique barriers to recruitment of Latinas and Hispanics exist including lack of awareness in research, lack of transportation, interference with work/family responsibilities, financial costs, negative side effects, and burdensome procedures, such as required time to participate, having multiple jobs, caring for more than one generation of family members (83). Regarding exercise-oncology research, specific barriers to exercise reported by LHBCS include lack of enjoyment, lack of knowledge on how to exercise, feeling self-conscious due to physical appearance, and discouragement (84). Therefore, there is a need for investigating culturally tailored exercise intervention approaches, such as that utilized in the ROSA trial, that are effective at reducing said barriers, improving survivorship outcomes, and maintaining feasibility for LHBCS (85).

We selected MetD as the primary outcome of the ROSA Trial, which has been an emerging concern particularly among breast cancer survivors (86). MetD can develop or worsen after curative treatment for many cancers including breast, prostate, and testicular, and can negatively affect cancer outcomes as well as overall health status (87, 88). Further, in relation to the development of MetD, higher rates of metabolic-related comorbidities have been observed in patients with breast cancer who had completed treatments, with obesity present in 51% of cases, hypertension in 34%, peripheral vascular disease in 26%, and diabetes in 13% (89). As breast cancer survival rates continue to improve worldwide (90), there is increasing focus on the role of modifiable risk factors on prognosis. For example, obesity at diagnosis (91), metabolic syndrome (92), and insulin resistance at diagnosis (93), as well as hyperinsulinemia post-treatment (11), are associated with increased risk of breast cancer mortality, all-cause mortality, and breast cancer recurrence. Notably, there are ethnic disparities where the risk of developing MetD is 50% higher among Latinas and Hispanics than other ethnic groups (14, 94), increasing the need to reduce this co-morbidity in this minority population (95, 96). Although the presence of MetD specifically in LHBCS has not been reported either prior to diagnosis or during survivorship, our previous study showed that metabolic syndrome and central obesity were highly prevalent in LHBCS with 100% of LHBCS (n=40) with either metabolic syndrome or central obesity assessed within six months following completion of cancer-related treatments (22, 23). Therefore, the findings of the ROSA trial in exercise-induced changes in MetD are expected to provide significant evidence to reduce MetD in LHBCS. Reducing MetD in LHBCS may ultimately improve mortality and cancer recurrence rates, if the appropriate intervention is employed and sustained.

In addition to contributing to the scarce literature in health disparities in minorities, this randomized controlled trial also uses a novel approach with a virtually supervised home-based exercise intervention *via* Zoom to increase participant outreach and due to the COVID-19 pandemic. Winters-Stone et al. (97) found better adherence and retention rates with virtually supervised exercise compared with in-person exercise sessions. To our knowledge, the ROSA trial is the first study using this exercise approach in minority cancer survivors. Advantages of virtually supervised exercise interventions include the opportunity to increase accessibility reducing travel time and cost burden, decreasing non-essential person-to-person contact, reaching participants in rural settings, and maintaining an intact individualized exercise program not often utilized with telephone- or test-based interventions (98). Therefore, our findings will allow us to determine if this exercise approach may be effective to improve health outcomes and if it reduces barriers to participation in exercise trials in this specific population (84, 99).

There are several strengths of the ROSA trial. We use a randomized controlled trial design in a specific cancer minority population of LHBCS, focusing on high-risk breast cancer survivors with high rates of inactivity. Moreover, our exercise intervention is rigorously developed incorporating progressive combined aerobic and resistance training and is delivered virtually in Spanish or English by certified clinical exercise physiologists to enhance participants’ convenience and potentially adherence. There are several limitations of the ROSA trial. Our supervised intervention setting, despite the virtual format, may not be highly scalable into community settings due to the costs involving home exercise equipment and securing exercise trainers for supervised exercise sessions. Furthermore, we measure several outcomes, which may increase Type I error risk. Given the nature of an exercise intervention there is an intrinsic impossibility to blind participants and study stuff after randomization. Although our follow-up period would allow us to determine whether metabolic health status can be maintained during a 4-month follow-up, this short-period, along with our smaller sample size, would not permit us to capture if our exercise intervention may be associated with reductions in comorbidities, recurrence, or mortality. Lastly, our population of LHBCS is only representative of the New England area and does not represent LHBCS nationally or globally.

In conclusion, the ROSA trial will contribute to understanding a significant gap in the literature seeking to diversify exercise oncology research by targeting a cancer minority of LHBCS and by examining whether exercise is effective in improving MetD and whether these improvements are maintained post-intervention in LHBCS. Findings from this trial will help to understand how exercise may impact health-related outcomes that might translate into survival benefits. Lastly, understanding the barriers to participation in exercise clinical trials in underrepresented cancer populations may help the development of future exercise oncology guidelines and public health recommendations in physical activity with specific information for cancer minorities, thus reducing health disparities.

## Ethics Statement

The research protocol involving human participants were reviewed and approved by the Dana Farber Cancer Institute Institutional Review Board (IRB#20-221). Written informed consent will be obtained from participants to participate in the study. This clinical trial is registered in ClinicalTrials.gov (NCT04717050).

## Author Contributions

CD-C conceptualized and designed the study. All authors contributed to the draft of the manuscript. CD-C acquired funding. All authors contributed to manuscript revision and approved the submitted version.

## Funding

Research Grant No. 131656-RSG-18-023-01-CPPB from the American Cancer Society. This funding source had no role in the design of this study and will not have any role during its execution, analyses, interpretation of the data, or decision to submit results.

## Conflict of Interest

Author FF was employed by the company Gerson Lehrman Group, LLC.

The remaining authors declare that the research was conducted in the absence of any commercial or financial relationships that could be construed as a potential conflict of interest.

## Publisher’s Note

All claims expressed in this article are solely those of the authors and do not necessarily represent those of their affiliated organizations, or those of the publisher, the editors and the reviewers. Any product that may be evaluated in this article, or claim that may be made by its manufacturer, is not guaranteed or endorsed by the publisher.
